# Alzheimer’s disease: targeting the glutamatergic system

**DOI:** 10.1007/s10522-020-09860-4

**Published:** 2020-02-11

**Authors:** Myra E. Conway

**Affiliations:** 1 Faculty of Health and Applied Sciences, University of the West of England, Coldharbour Lane, Bristol, BS16 1QY UK; 2 Faculty of Health and Life Sciences, University of the West of England, Coldharbour Lane, Bristol, BS16 1QY UK

**Keywords:** Glutamate, Aging, Alzheimer's disease, BCAT, Branched chain amino acids

## Abstract

Alzheimer’s disease (AD) is a debilitating neurodegenerative disease that causes a progressive decline in memory, language and problem solving. For decades mechanism-based therapies have primarily focused on amyloid β (Aβ) processing and pathways that govern neurofibrillary tangle generation. With the potential exception to Aducanumab, a monotherapy to target Aβ, clinical trials in these areas have been challenging and have failed to demonstrate efficacy. Currently, the prescribed therapies for AD are those that target the cholinesterase and glutamatergic systems that can moderately reduce cognitive decline, dependent on the individual. In the brain, over 40% of neuronal synapses are glutamatergic, where the glutamate level is tightly regulated through metabolite exchange in neuronal, astrocytic and endothelial cells. In AD brain, Aβ can interrupt effective glutamate uptake by astrocytes, which evokes a cascade of events that leads to neuronal swelling, destruction of membrane integrity and ultimately cell death. Much work has focussed on the post-synaptic response with little insight into how glutamate is regulated more broadly in the brain and the influence of anaplerotic pathways that finely tune these mechanisms. The role of blood branched chain amino acids (BCAA) in regulating neurotransmitter profiles under disease conditions also warrant discussion. Here, we review the importance of the branched chain aminotransferase proteins in regulating brain glutamate and the potential consequence of dysregulated metabolism in the context of BCAA or glutamate accumulation. We explore how the reported benefits of BCAA supplementation or restriction in improving cognitive function in other neurological diseases may have potential application in AD. Given that memantine, the glutamate receptor agonist, shows clinical relevance it is now timely to research related pathways, an understanding of which could identify novel approaches to treatment of AD.

## Introduction

Dementia is defined as a loss in memory or brain function that affects an individuals day-to-day living, where they present with a range of symptoms including memory loss, difficulty with problem solving or language. According to the World Health Organisation, there is an estimated 46.8 million people worldwide living with dementia that is predicted to almost double every 20 years. The types of dementia vary with respect to the areas of the brain affected, related symptom patterns and the underlying pathology that include (but are not limited to) Alzheimer’s disease (AD), Vascular dementia, mixed dementia, dementia with Lewy bodies and Frontotemporal dementia (reviewed in Alzheimer’s Association 2016). AD, which reflects about 64% of cases, is a progressive neurodegenerative disorder characterised by the accumulation of extracellular amyloid β peptide (Aβ) and intracellular neurofibrillary tangles (NFTs) associated with extensive neuronal cell death and loss of brain volume. *N*-methyl-d-aspartate receptor (NMDAR) activation and related excitotoxic events that lead to synaptic dysfunction has also been associated with AD pathology (Liu et al. 2019). The aetiology of AD is not well understood, but is often characterised as early-onset familial AD (less than 65 years of age) and late onset AD (greater than 65 years of age) (Tellechea et al. 2018; Wattmo and Wallin 2017). Diagnosis is therefore confounded by the complexity of disease pathology and classification, where the incidence and prevalence increases significantly with age. Confirmation of AD can only be given at autopsy when morphological and histological examinations have been validated.

Whilst defective amyloid precursor protein (APP) processing and Aβ aggregation more than likely contribute to AD pathology the number of plaques generated do not always reflect disease severity, indicating that other factors, such as genetics and environmental cues, have a role in disease progression. Neuronal loss has also been related to the dysregulation of the cholinergic system and glutamatergic system (Ferreira-Vieira et al. 2016). Deficits in these systems can influence memory, cognition and behaviour, including cortical and hippocampal processing. Currently, prescribed treatments for AD are based on cholinesterase inhibitors (galantamine, rivastigmine and donepezil) or glutamatergic targets (memantine) (Table 1). Whilst these treatments offer some reprive to symptoms, the improvement is modest and temporary, indicating that like Aβ targeted approaches, the heterogenity of the disease calls for a stratified approach for effective treatment. Based on genome-wide association studies, over 20 genetic risk factors have been suggested to feature strongly in AD pathology e.g. those carrying the APOE4 allele are considered to be at higher risk of developing AD. Factors that regulate metabolism in particular glucose and cholesterol metabolism together with autophagy-related recycling pathways and inflammatory responses involving microglial activation show wide genetic and epidemiological correlations. Hypertension, vascular risk factors and diabetes are also considered to adversely influence risk (reviewed in Lane et al. 2018).

**Table 1:** Current FDA approved drugs to treat AD

Drug	Trade name	Mode of action	Treatment stage
Donepezil	Aricept	Acetylcholinesterase inhibitor^a^	All stages
Galantamine	Razadyne	Acetylcholinesterase inhibitor^a^	Mild to moderate
Rivastigmine	Exelon	Acetylcholinesterase inhibitor^a^	Mild to moderate
Memantine	Namenda	NMDA receptor antagonist	Mild
Donepezil + memantine	Namzaric	Acetylcholinesterase inhibitor + NMDA receptor antagonist^b^	Moderate to severe

^a^May delay or slow worsening of symptoms. Effectiveness varies from person to person

^b^Effective in individuals with moderate to severe AD who are taking a cholinesterase inhibitor might benefit by also taking memantine

In this review, we discuss how brain glutamate (Glu) levels change as we age, how metabolism and nutrition regulate this neurotransmitter and how excitotoxicity causes neurodegeneration. We highlight the importance of Glu regulation in pre-synaptic neurons and how anaplerotic pathways governed by aminotranferase proteins, such as the branched chain aminotransferase (BCAT), play an integral role in regulating brain Glu. We offer insight into how these proteins and the branched chain amino acids (BCAAs) may present novel targets to delay the progression of AD.

### The amyloid and Tau hypothesis

**Fig. 1:**
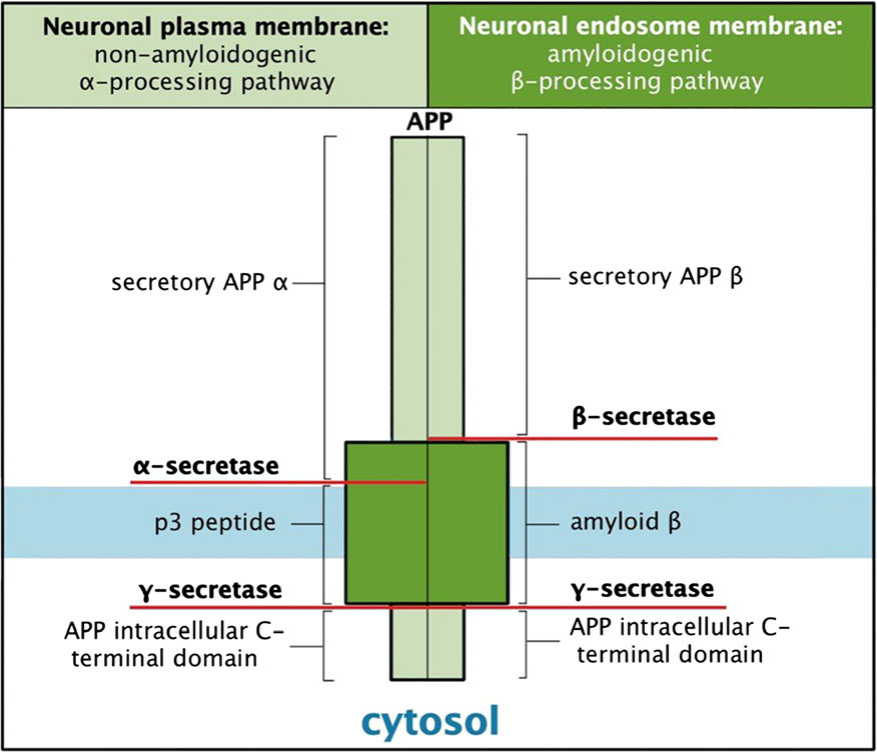
Amyloid precursor protein processing pathways.APP undergoes either α or β secretase processing generating the secretory APPα subunit or the secretory APP β subunit, respectively. Subsequent cleavage by γ-secretase generates the p3 peptide and Aβ for the amyloidogenic and non-amyloidogenic pathway, respectively. (Moraes and Gaudet 2018) with permission from Oxford University Press

The search for mechanism-based therapies has focussed on the histopathological and morphological changes and their related effect on cognitive decline. Early-onset familial AD caused by mutations in the APP gene or in genes of proteins important to APP processing, including presenilin 1 (PSEN1) and presenilin 2, support the hypothesis that defective APP processing is a key factor in AD pathology (Reitz et al. 2011; Nizzari et al. 2012). Processing of the APP by α, β, and γ secretases can be summarised in the non-amyloidogenic and amyloidogenic pathway (Fig. 1) (reviewed in O’Brien and Wong 2011; Tiwari et al. 2019). In brief, the non-amyloidogenic pathway generates soluble APPα, the non-toxic peptide P3 and the APP intracellular domain. In neurons, A Disintegrin and metalloproteinase domain-containing protein 10 (ADAM10) and ADAM17 are considered the major α-secretases that cleave within the Aβ domain. In the amyloidogenic pathway, β-site APP cleavage enzyme 1 (BACE-1) cleavage occurs at the N-terminus of the Aβ peptide generating APP-β and β C-terminal fragment (β-CTF). This is followed by γ-secretase cleavage generating the Aβ peptide. The γ-secretase protease is composed or four individual proteins: PSEN1, nicastrin, anterior pharynx defective 1 (Aph1), and presenilin enhancer 2. The resulting Aβ [Aβ (1–40) and Aβ (1–42)] peptides can aggregate to form oligomers that are found in senile plaques evident in AD brain. Associated neurotoxic events include generation of ROS, loss of mitochondrial function (Reddy and Beal 2008), disruption of calcium homestasis together with neuroinflammation due to microglia activation by Aβ (Cai et al. 2014). Decades of clinical trials dedicated to target Aβ processing have failed to generate a treatment for AD (Tolar et al. 2019). The most recent trial with Aducanumab (BIIB037), a monoclonal antibody that targets Aβ, failed to reach primary endpoint targets in March, 2019, where an indepdendent data monitoring committee determined that there was insufficient evidence to support drug efficacy. However, in this study, two Phase III trials, ENGAGE and EMERGE were running consecutively, where EMERGE was later shown to be ‘trending postive’. Subsequent analysis of this larger data set with a focus on the outcome of people on the highest aducanumab dose showed a significant reduction in decline on the primary endpoint but also on secodnary endpoints such as MMSE and ADAS-Cog. These discrepancies were reportedly due to a change in the trial protocols. Based on these outcomes, Biogen plan to apply for regulatory approval in the US in 2020. Should this succeed this will be the first therapeutic agent to target Aβ and show promise in the treament of AD.

The second major molecular pathway identified as a therapeutic target is based on the tau protein, which has a microtubule binding domain and plays a critical role in tubulin assembly and stabilisation of microtubules (Duan et al. 2017). In AD, hyperphosphorylation of tau results in a conformational change that causes microtubule destabilisation and aggregate accumulation forming NFTs. When microtubule dynamics are dysregulated, neuronal integrity is compromised and degeneration follows. This is further precipitated by the aggregation of tau generating NFTs, which is thought to block axonal and dendritic transport in neurons. Accumulation of Aβ and NFTs has associated negative effects on neurotransmitter systems such as the Cholinergic and Glutamatergic system that impair long-term potentiation and synaptic plasticity (Ferreira-Viera et al. 2016; Benussi et al. 2019; Hynd et al. 2004).

## Brain glutamate

### Glutamate in the brain, aging and AD-related changes

Glutamate is one of the major excitatory neurotransmitters in the mammalian brain, important in memory, learning, cognition, motor behaviour as well as the immediate precursor to GABA and glutathione (Danbolt 2001). Under normal physiological conditions Glu plays a role in dendrite and synapse formation. Glutamatergic activity can be measured using proton magnetic resonance spectroscopy (^1^H MRS) (Hall et al. 2012; Bookheimer et al. 2000), that is reflective of neuronal integrity. The use of ^1^H MRS has also emerged as a promising tool in neurological and psychiatric disorders including the investigation of AD (Wang et al. 2015a, b; Su et al. 2016) and ALS (Kalra 2019). Age-related changes in selected ^1^H MRS biomarkers have been reported, where lower concentrations of Glu recorded in the motor cortex positively correlated with *N*-acetyl aspartate (NAA) and creatinine (Cr) consistent with neuronal loss/shrinkage with age (Kaiser et al. 2005). Until recently, measurement of GABA using ^1^H MRS was less reliable, but it has been shown that like Glu it declines with age in the human brain (Gao et al. 2013).

Furthermore, ^1^H MRS was found to improve prognostic accuracy for conversion from mild cognitive impairment (MCI) to dementia (Modrego et al. 2005) and even from cognitive health to MCI (Kantarci et al. 2017). A meta-analysis by Wang et al. reported a significant reduction in NAA in the posterior cingulate (p < 0.005) and bilateral hippocampus (p < 0.005) in AD patients (Wang et al. 2015a, b). A decrease in the NAA/Cr ratio together with an increase in the mI/Cr ratio was also observed, indicating that collectively these markers hold promise as biomarkers of brain function and more importantly evidence that the glutamatergic pathway is important in AD pathology. Concentrations of Glu were also shown to be lower in the posterior cingulate cortex of AD patients, either when measured alone or as a combined measure of Glu+glutamine (Glx) (Antuono et al. 2001; Fayed et al. 2011; Rupsingh et al. 2011). Related studies showed that a combination of Choline/Cr, Glx/Cr, and NAA/Cr ratios in the posterior cingulate cortex successfully differentiated between carriers from non-carriers of a fully penetrant, early onset familial (PSEN1 gene) mutation at different disease stages (Londono et al. 2014). However, few studies report the measurement of Aβ aggregates (measured by positron emission tomography) and their association with ^1^H MRS (Kantarci 2007). In a pilot study, GABA, Glx and NAA were assessed in the posterior cingulate cortex relative to cognitive assessments, Aβ deposition, and APOE genotype of healthy control compared with amnestic MCI (aMCI) subjects (Riese et al. 2015). Here, levels of these biomarkers were significantly reduced in aMCI and correlated with CERAD (The Consortium to Establish a Registry for Alzheimer's Disease) word learning performance. Although, a correlation between GABA and Glx was not associated with Aβ deposition or APOE genotype, further longitudinal studies are warranted as the prognostic value of these biomarkers relative to Aβ or conversion to AD may hold promise. The authors also reflected that this does not rule out potential correlations between these biomarkers and Aβ load in other brain areas such as the hippocampus. Together, ^1^H MRS studies confirm that glutamatergic neurotransmission decreases as we age but is further perturbed in individuals with aMCI and AD, respectively and correlates with cognitive measures.

### Glutamate/glutamine cycle

**Fig. 2:**
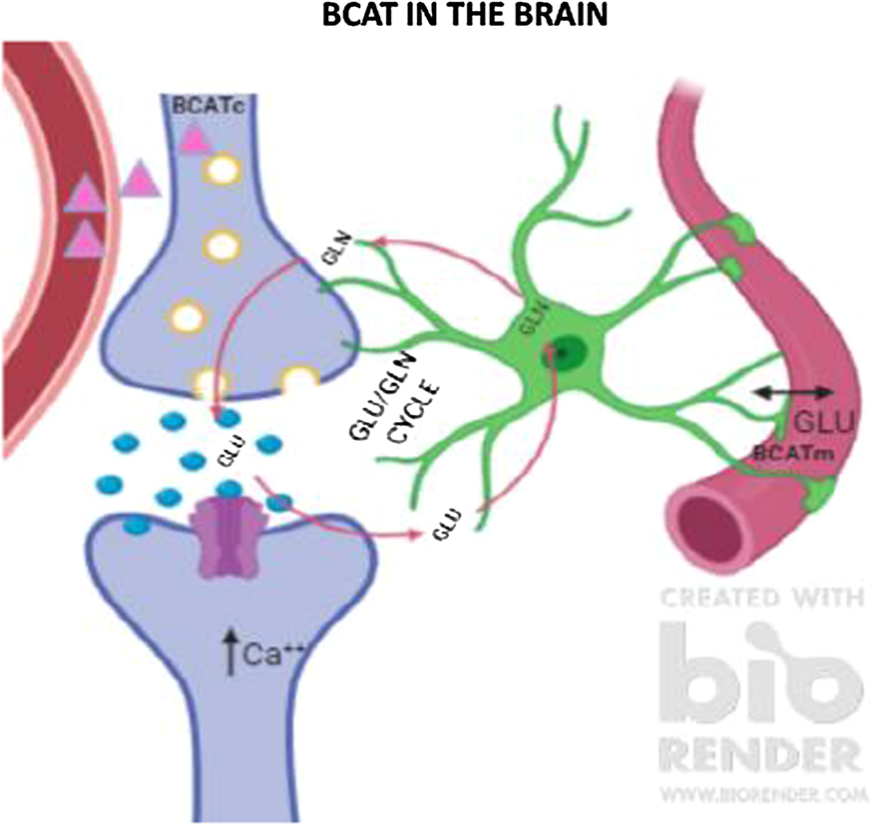
BCAT metabolism in the human brain. Leucine (∆) readily crosses the blood brain barrier and undergoes BCATc transamination in neuronal cells producing glutamate. Excess glutamate generated during excitation is taken up by astrocytes, where glutamate is regenerated through the glutamate/glutamine cycle. Glutamate may also be removed by endothelial cells of the vasculature. (Image generated using BioRender)

Glutamate is extensively distributed throughout the CNS (Collingridge and Lester 1989). Regulation of brain Glu is thought to be primarily governed through the Glu/glutamine cycle, where excess Glu remaining after excitation is taken up by astrocytes (Fig. 2) (reviewed in Conway and Hutson 2016; Yudkoff 2017). At rest, the concentration of Glu in the synaptic cleft is around 0.6 μM (Bouvier et al. 1992) but debate exists as to the actual concentration (reviewed in Fetherstone and Shippy 2008). Following presynaptic neuronal depolarisation, synaptic vesicles that store glutamate, first fuse with the membrane and then release Glu into the synapse. Glutamate can then activate a variety of ionotropic and metabotropic receptors on postsynaptic and presynaptic neurons as well as glial cells. During excitation the level of Glu increases to 10 μM (Clements et al. 1992), where overstimulation is prevented by the rapid and efficient removal from the extracellular space. This is facilitated by astrocytes that express high levels of the Glu specific transporter (GLAST/EAAT1 and GLT1/EAAT2). Glutamate is converted through amidation to glutamine by the microsomal enzyme glutamine synthetase (restricted to astrocytes). As glutamine is non-neuroactive it can be released into the ECF for reuptake by the pre-synaptic neuron. This then replenishes the store of Glu through deamidation by mitochondrial phosphate-dependent glutaminase and packaged into synaptic vesicles for reuse. Whilst the glutamate/glutamine cycle is an efficient method to regenerate Glu, this recycling is not 100% effective. During its transition some Glu is ‘lost’ in astrocytes through metabolism, generating lactate (Sonnelwald et al. 1993), or in the production of purines and glutathione (Shank et al. 1985). As a result, anaplerotic pathways must interface with the Glu-glutamine cycle to regenerate this ‘lost’ Glu necessary to sustain efficient neurotransmission. This is in part facilitated by pyruvate carboxylase (Gamberino et al. 1997), an enzyme found solely in astrocytes that utilizes brain CO_2_ to replenish the carbon required for the TCA cycle, which as a result contributes to the overall concentration of glutamine produced (Oz et al. 2004). A limiting factor in this reaction is the source of nitrogen, where the BCAAs, aspartate and more recently alanine serve as potential nitrogen donors (Hutson et al. 1998, 2001; Lieth et al. 2001; Bixel et al. 1997, 2001; Yudkoff et al. 1983, 1996; Kanamori et al. 1998). The regulation of brain Glu is important as uptake from the blood is minimal, however, precursors to its synthesis such as the BCAAs are easily taken up, in particular leucine (Oldendorf et al. 1977; Smith et al. 1987). The BCAAs are metabolised through a series of reactions involving BCAT and the rate limiting oxidation step catalysed by the branched chain α-keto acid dehydrogenase (BCKD) complex.

### The branched chain aminotransferase proteins

**Fig. 3:**
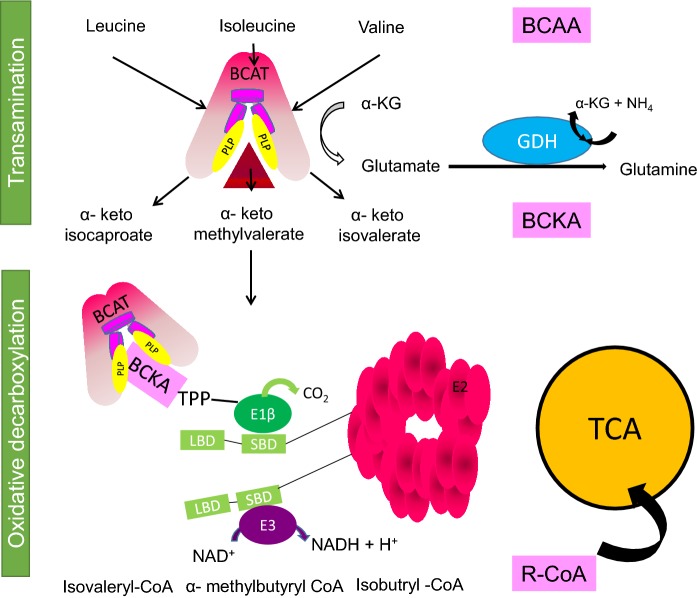
Branched chain amino acid (BCAA) oxidation. Transamination of the BCAAs, leucine, isoleucine and valine with α-keto glutarate forming the respective α-keto acids, (BCKAs: α-ketoisocaproate (KIC), α-keto-β-methylvalerate (KMV) and α-ketoisovalerate (KIV)) and glutamate, regenerating the enzyme. Subsequent oxidation by the branched chain α-keto acid dehydrogenase complex generates the branched chain acyl CoA, which enter the TCA cycle. Glutamate can be further metabolised by glutamate dehydrogenase to generate glutamine. (Adapted from Conway and Hutson 2016)

The canonical role of the BCAT proteins is the transamination of the BCAAs, leucine, isoleucine and valine with α-keto glutarate to form Glu and their respective branched chain α-keto acids (BCKA) (α-ketoisocaproate (KIC), α-keto-β-methylvalerate (KMV) and α-ketoisovalerate (KIV)) regenerating the pyridoxal phosphate (PLP) form of the enzyme (Ichihara and Koyama 1966) (Fig. 3). Complete oxidation of the BCAAs is facilitated through the BCKD complex (Harris et al. 2004). Glutamate dehydrogenase (GDH), a mitochondrial matrix enzyme, is responsible for the oxidative deamination of Glu to α-KG and free NH_4_^+^, using either NAD^+^ or NADP^+^ as a co-factor. The BCATm:BCKD complex and BCATm:GDH form metabolons, which facilitate effective substrate channelling (Islam et al. 2007, 2010; Hutson et al. 2011). Whole body tissue expression and compartmentation of these proteins drives intra- and inter-organ exchange of nitrogen and carbon (Hutson and Harper 1981). Unlike all other amino acids, the BCAAs do not undergo transamination in the liver but are directed to skeletal muscle, one of the main sites of BCAA uptake. Because skeletal muscle transaminase activity exceeds the oxidative capacity, BCAAs act as nitrogen donors in peripheral metabolism (Hutson et al. 1978). In the central nervous system transamination and oxidation also exhibit cell-specific localization and participate in nitrogen and carbon shuttles (Hutson et al. 1998). For the purpose of this review we will focus on the role of the BCAT protein in the brain, in particular with respect to the regulation of brain Glu.

### Cellular distribution of branched chain aminotransferases

The basis for regulating brain Glu originates in part due to the distinct subcellular location and tissue specificity of the BCAT proteins (Daikin and Yudkoff 2000). These enzymes are indispensable and have been reported in most tissues with varied expression levels. Of those studied to date there are two main isoforms, cytosolic, BCATc and mitochondrial, BCATm, encoded by two different genes. The cytosolic form has been identified primarily from brain, placenta and ovary (Hall et al. 1993; Hutson 1988; Hutson et al. 1998; Sweatt et al. 2004a, b; Garcia-Espinosa et al. 2007). The mitochondrial isoform, is responsible for the majority of transamination outside the central nervous system. The highest levels of BCATm recorded are in the pancreas, kidney, stomach and brain (Suryawan et al. 1998). Only variants of BCATm have been described, a novel alternatively spliced PP18b variant found in placental tissue (Lin et al. 2001) and a novel co-repressor for thyroid hormone nuclear receptors (P3) (Than et al. 2001). Although the biological significance of these variants remains to be determined, P3 has been shown to act as a corepressor for thyroid hormone nuclear receptors. More recently, a clinically relevant mutant form of BCATm has been reported, which has homozygous or compound heterozygous *BCAT2* mutations, discussed in Mutations of BCAT leads to mild cognitive impairment and brain lesions Section (Wang et al. 2015a, b; Knerr et al. 2019).

The distribution of BCAT in the brain was first characterised in rat and murine models (Oldendorf 1977; Bixel et al. 1997; Bixel et al. 2001; Sweatt et al. 2004a, b; Garcia-Espinosa et al. 2007). Our group subsequently mapped BCATc to the human brain, where it was found in all brain regions and consistent with the rat model was neuron-specific, with strongest labelling reported in the parietal cortex (Hull et al. 2012). The hippocampus showed intense immunoreactivity for BCATc of pyramidal cells (approximately 70% of neuronal cells) in the pyramidal cell layer, with moderate BCATc staining in the cell bodies of interneurons (GABAergic or glycine). Pyramidal cells are the primary cells lost from the neocortex in AD (Bussiere et al. 2003). The cell bodies of glutamatergic cells within the temporal and hippocampus showed intense staining relative to the dendrite regions, reflecting the possibility that their primary role would be to contribute to the Glu metabolic pool used to generate Glu rather than the Glu pool used during excitation. Conversely, in the areas of the supraoptic tract intense staining along axons was noted indicating an additional role of BCATc transamination in Glu release in this region. In the cerebellar cortex and granular cell layer, GABAergic neurones including stellate, basket and Golgi neurons within the molecular layer were positive for BCATc. The distribution and the varied intensities of BCATc expression in the hippocampus, where staining of the CA3 region was more intense than the CA1 region, mirrored the findings of Castellano et al. (2007), who investigated mRNA expression of BCATc in postnatal and adult brains of mice. This pattern of BCATc staining was reported throughout the human brain evidencing that BCATc is important in glutamatergic and GABAergic neurotransmission.

**Fig. 4:**
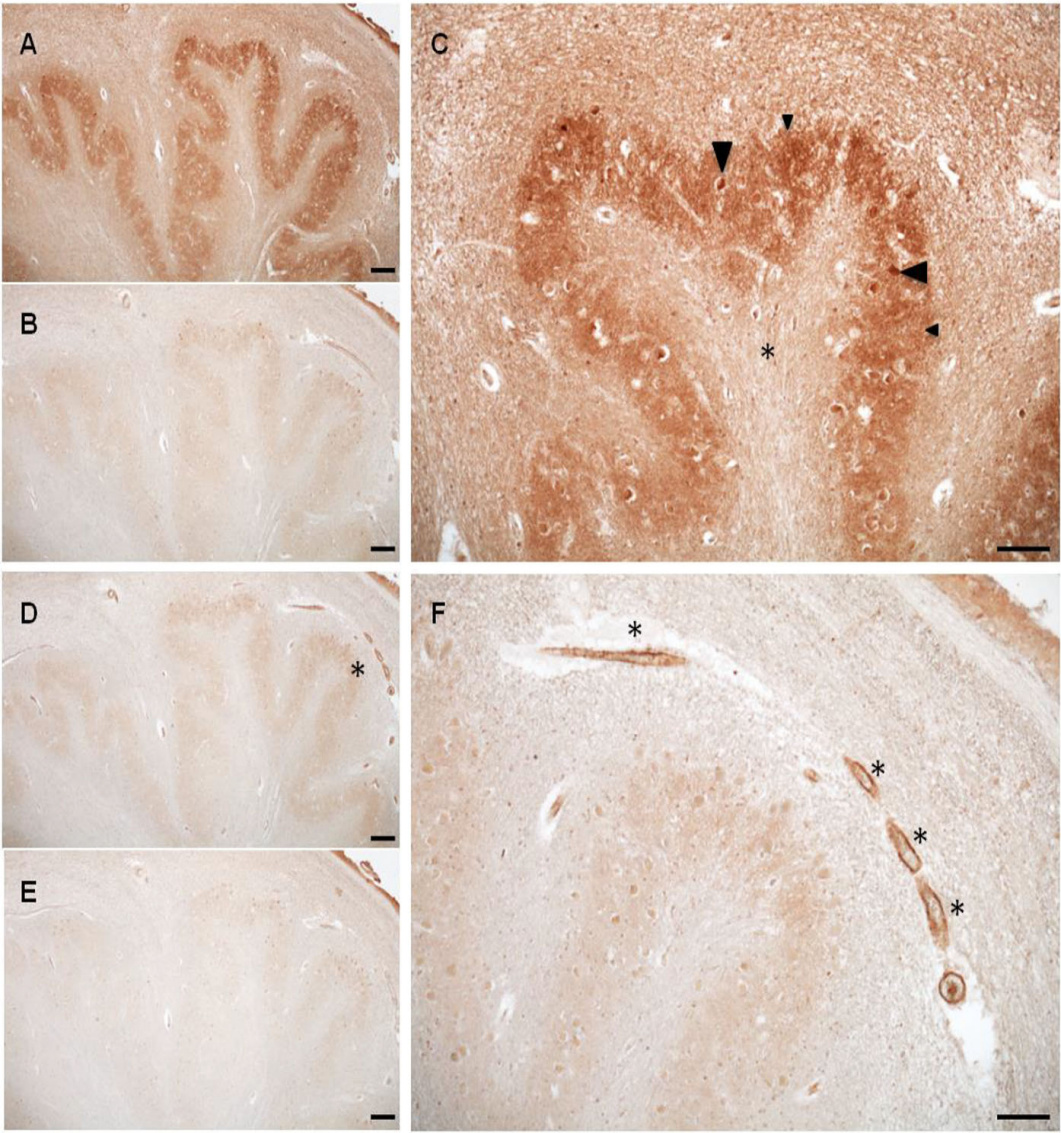
Medulla BCATc and BCATm staining. **a** BCATc staining of the inferior olivary nucleus. **b** Antigen incubation of serial section, at 200× molar excess. **c** Increased magnification of the inferior olivary nucleus showing staining of small neurons (large arrow) and neuropil staining (small arrow) along with immunonegative hylum (*). **d** BCATm staining of the inferior olivary nucleus. **e** Antigen incubation of serial section, at 200× molar excess. **f** Vessel staining (*) within the amiculum of the inferior olivary nucleus. Scale bar: **a**, **b**, **d** and **e** 200 μm; **c** and **f** 100 μm. (Hull 2012)

What was clear was that wherever BCATc staining occurred BCATm was absent and vice versa (Fig. 4). Immunopositive staining for BCATm was vessel and capillary in nature. This staining was evident in all major anatomical regions of the brain assessed with the exception of the parietal lobe and medulla. Examples of areas positive for BCATm staining include the cerebral cortex, subdivisions of the basal ganglia and the diencephalon, deep nuclei and the hippocampal formation. The endothelium of capillaries and larger blood vessels were immunopositive for BCATm and showed clear, punctate staining indicative of mitochondria. The role of endothelial cells in regulating blood Glu has been researched in-depth, with a focus on transporters, which regulate Glu exchange at the blood brain barrier (O’Kane et al. 2004). Glu entry from the peripheral system to the brain is minimal, which prevents neurotoxicity, whereas brain Glu efflux is facilitated by the EAAT transporter (Chaudhry et al. 1995; O’Kane et al. 1999; Hosoya et al. 1999; Gottlieb et al. 2003; Uchida et al. 2011), the rate of which is considered to be influenced by blood Glu (Gottlieb et al. 2003; Zlotnik et al. 2008, 2012; Teichberg et al. 2009). Until the study by Helms et al. (2012), the potential for Glu metabolism within endothelial cells was not considered. Our group have shown that GDH and the BCKD complex are also expressed in endothelial cells, indicating that Glu oxidation can occur and will dependent on concentration and cellular redox state (Hull et al. 2018). The role of BCATm in endothelial cells has yet to be confirmed but most likely will be important in regulating brain glutamate through metabolism through the BCATm/BCKD/GDH metabolon or additional cellular roles yet to be defined.

### The role of BCAT in brain metabolism.

Dietary BCAAs play a significant role as nitrogen donors in brain metabolism. Tracing studies of [^15^ N] leucine in rodent models showed that BCAAs were metabolised faster than they were incorporated into proteins in brain slices (Chaplin et al. 1976). Approximately 30% of the nitrogen of Glu/glutamine was derived from leucine alone (Brand 1981; Brand and Hauschildt 1984), which was later supported using in vivo rat brain and ex vivo rat retina models (accepted models of Glutamatergic neurons) (Lieth et al. 2001; LaNoue et al. 2001). Based on the de novo synthesis of Glu from leucine, together with the location of BCATm in astrocytes and BCATc in neuronal cells, it was proposed that an exchange of metabolites between these cells, governed by BCAT transamination, supports Glu synthesis and regulation of the Glu/glutamine cycle (Yudkoff et al. 1997; Hutson et al. 1998; Hutson et al. 2001). In this rat model, termed the “BCAA/BCKA shuttle”, it was proposed that the distribution of BCATm to astrocytes and BCATc to neuronal cells indicated a role for transamination of BCAAs in astrocytes, generating BCKAs, which are thought to be taken up by neuronal cells to regenerate the ‘lost Glu’ (Hutson et al. 2001; Yudkoff 2017). However, as discussed, in human brain BCATm was found in endothelial cells indicating that an alternative model needs to be considered for human brain glutamate regulation.

In human brain, we propose that in neuronal cells BCAAs are transaminated by BCATc generating Glu, which contributes both to the pool of neurotransmitters and the Glu required for release during excitation. As described, Glu can be taken up by endothelial cells, which can be further metabolised or released into the blood, dependent on the serum Glu concentration. Metabolism of Glu would release α-keto glutarate that could enter the TCA cycle. The respective BCAA, could be further oxidised for energy or be taken up by neuronal cells to regenerate the ‘lost’ Glu. We propose that the expression of BCATm, the BCKD complex and GDH offer an auxiliary mechanism to regulate brain Glu, in particular under conditions where the level of Glu is high, offering a neuroprotective role in the first instance (Hull et al. 2015a, b). However, in disease conditions such as AD, Glu toxicity resulting in neuronal cell death indicates that there is a failure in this mechanism to effectively remove toxic levels of Glu. Here, a role for BCAT regulation through dietary serum BCAAs may be important.

## Glutamate, BCAA toxicity and neurodegeneration

### Glutamate excitotoxicity

Damage to glutamatergic neurons, in particular cell bodies and neurites in layers III and IV of the neocortex, together with damage to glutamatergically-innervated cortical and hippocampal neurons, are particularly evident in AD brain (Albin and Greenamyre 1992). Altered expression of glutamatergic transport and receptors were also reported in sporadic AD (Jacob et al. 2007), where a role for astrocyte transporters were shown to be involved with Aβ-induced synaptic dysfunction (Huang et al. 2018). Whilst an important neurotransmitter, pathological accumulation of Glu results in this amino acid becoming a potent neurotoxin (Choi et al. 1987; Mattson and Chan 2003). This is in part due to the time-related exposure, overstimulating the post synaptic response causing an increase in the entry of calcium into neurons (Sattler and Tymianski 2000; Mattson and Chan 2003). Several events have been linked with excitotoxicity through the NMDAR. First, the swelling of cell bodies and dendrites causes depolarisation, which depends on the extracellular concentrations of Na^+^ and Cl^+^ (Rothman 1985). The second event is marked by slow neuronal degeneration mediated by Ca^++^ influx (Koh and Choi 1991). In vitro studies in which hippocampal neurons are exposed to toxic levels of Glu, Ca^++^ levels remained elevated for about 1 h before returning to pre-stimulus levels (Dubinsky 1993), however, over 24 h 84% of neurons expired. As calcium signalling regulates a host of cellular events the consequence of Ca^++^ overload involves activation of catabolic enzymes including calpain I (Siman and Noszek 1988), phospholipases (Lazarewicz et al. 1990) and arachidonic metabolism (Chan and Fishman 1978). This results in an increase in reactive oxygen and reactive nitrogen species and neuronal cellular collapse through cytoskeletal breakdown and membrane degeneration. Elevated Ca^++^ may also activate protein kinases that contribute to hyperphosphorylation of tau and ubiquitin (Mattson 1992). More recently, glutamate toxicity has been asscoiated with tau-mediated neuronal cell death and behavioural deficits in drosophila (Killian et al. 2017). NMDAR antagonists such as memantine block this receptor and can reduce the influx of Ca^2++^. Treatment using memantine has improved cognition, behaviour, global function but the degree of efficacy remains to be fully determined (Kishi et al. 2017; Matsunga et al. 2015). Although it is evident that the release of large concentrations of Glu into the ECF can occur under pathogenic conditions, the source of this Glu has been met with extensive debate. However, given that the highest concentration of Glu is stored in neuronal rather than glial cells, glutamatergic neurons in particular are considered the most likely source.

### Regional increase of BCAT in AD brain—Glu toxicity.

**Fig. 5:**
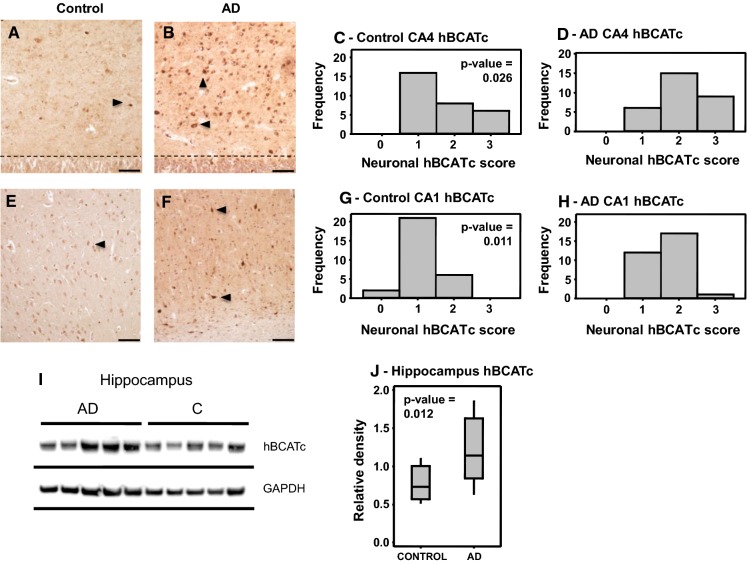
Increased hBCATc expression in the hippocampus of AD brains. CA4 region of the hippocampus in a control (**a**) and AD brain (**b**) showing intensely labelled neurons (large arrows) and the granule cell layer (dotted line). CA1 region of the hippocampus in a control (**e**) and AD (**f**) subject showing intensely labelled neurons (large arrows). **c**, **d**, **g**, **h** The slides were scored on a 0–3 scoring system and analyzed for significance using the Wilcoxon-Mann-Whitney test in Minitab™ as described in materials and methods. **i** Western blot analysis of hippocampal tissue. The density of each band was measured using ImageJTM software (Wayne Rasband, National Institute of Health, USA) and analyzed for significance using a one-way ANOVA test in Minitab™. **j** Interquartile range (box) sample variability (whiskers) and the median (horizontal line within the interquartile range) are shown. Magnification for **a**, **b**, **e**, and **f**, ×10. Scale bar: 200 μm. (Reprinted from Hull et al. (2015a) with permission from IOS Press)

**Fig. 6:**
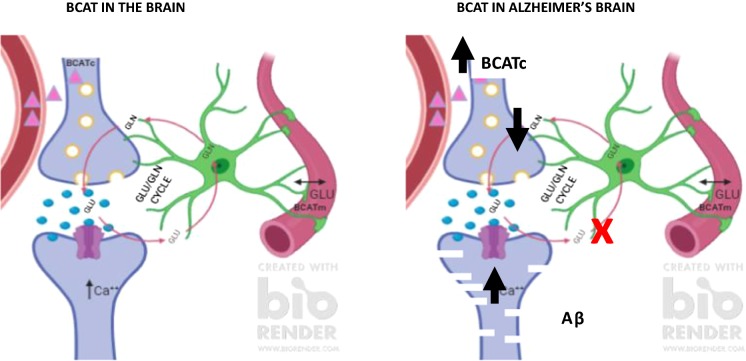
BCAT metabolism in the human brain. Under conditions where excitotoxicity persists, astrocytes are ineffective at clearing excess glutamate. This results in the post synaptic overstimulation of NMDA receptors causing calcium overload and ROS-related cell death. Moreover, levels of BCATc are increased in glutamatergic neurons, increasing the synthesis of glutamate, exacerbating neuronal toxicity. (Image generated using BioRender)

Given the high expression of BCATc in glutamatergic and GABAergic neurons and its role in regulating brain Glu, our group examined the levels of the BCAT isoforms in AD brain. Our focus was on the main regions of the brain affected by AD pathology where we found a regional increase in BCATc in the hippocampus (Hull et al. 2015a, b), specifically the CA1 and CA4 region, the area first targeted by AD pathology (Fig. 5). An increase in the level of BCATc could be considered a compensatory response to expand the Glu pool in response to the reduced uptake of synaptic Glu by astrocytes. We assigned a neuroprotective role to BCATc but proposed that should transamination in the direction of Glu synthesis persist it could exacerbate toxicity contributing to neuronal degeneration (Fig. 6). A neuroprotective role for BCATc has been reported in related animal models of brain injury. A study by Kholodilov et al. (2000) reported that following developmental striatal target injury, BCATc was upregulated in the substantia nigra of rat. However, this upregulation was evident in morphologically intact cells with no evidence of apoptotic features, supporting a role in cell survival. Upregulation of BCATc in rodent models in response to brain-derived neurotrophic factor (BDNF), subsequent to visual cortex ablation, was also associated with neuroprotection (Castellano et al. 2006). Moreover, in a subsequent study an increase in BCATc in response to BDNF was particular to the parietal cortex, the hilus and CA3 hippocampal subfield (Madeddu et al. 2004). These regional increases support a role for BCATc in neuronal survival in areas heavily populated with glutamatergic neurons and indicate that BCAT metabolic activity is important under conditions that threaten cell survival. Therefore, it is possible that in AD too the upregulation of BCAT is a physiological response, as in general the neurons that were immunopositive for BCATc in the human brain tissue also appeared morphologically intact. Importantly, the immunohistochemistry data, showed that the intensity of BCATc labelling in both cell bodies and dendrites was most pronounced in the hippocampus, a region that is known to be particularly susceptible to excitotoxic neuronal injury at an early stage of AD, highlighting that sustained increase in BCATc may contribute to injury. In addition, the anti-epileptic drug gabapentin, which inhibits BCATc, has proved successful in treating behavioural alterations in subjects with AD (Cooney et al. 2013; Supasitthumrong et al. 2019). A more widespread increase in the level of BCATm was also reported that extended throughout the frontal and temporal regions of AD brain. An increase in BCATm was also reported in cases with dementia with Lewy bodies and vascular dementia (Ashby et al. 2017). Similar to BCATc, we propose that increased levels of endothelial BCATm would serve in a neuroprotective capacity. As astrocyte uptake is poor the endothelial system may operate as an auxiliary mechanism to support glutamate efflux (Hull et al. 2015a, b). However, further studies are required to fully determine the impact of increased BCAT levels in AD brain.

More recently, we reported that serum BCAT was also higher in AD individuals relative to healthy age-matched controls (Hudd et al. 2019). In this study we adopted a combined approach to generate a differential diagnosis of people presenting with MCI that will progress to AD. To this end, we used a battery of cognitive assessments together with magnetic resonance imaging and serum analysis of BCAT, the BCAAs and the aromatic amino acids. Our data shows a substantive mutually correlated system between BCAT and glutamate, neurophysiological tests and magnetic resonance imaging for the diagnosis of AD. Our model showed that BCAT and glutamate accurately distinguish between control and AD participants and in combination with the neurophysiological test Montreal Cognitive Assessment, improved the overall sensitivity to 1.00 and specificity to 0.978. This study indicated that BCAT and glutamate hold promise as early indicators of disease pathology. However, a limitation to this study was the timeframe, where a longitudinal study would better define the utility of BCAT and glutamate as biomarkers.

### Moonlighting roles for BCAT

Although the canonical role for BCAT has traditionally been associated with transamination and glutamate regulation, it is important to note that it is also a redox-sensitive protein subject to oxidation (Conway et al. 2002), S-nitrosylation (Conway et al. 2004) and S-glutathionylation (Conway et al. 2008). Whilst the role of BCAT in regulating brain glutamate is established the importance of the redox switch has yet to be fully appreciated, in particular under conditions that affect the redox state of the cell. This redox switch is unique to these aminotransferases and has been shown to be important in regulating metabolite shuttling between the BCKD complex and GDH (Islam et al. 2007). This is inhibited through oxidation that not only inactivates BCAT but also destabilises the metabolic complexes (Islam et al. 2010). Targeted proteomic studies have indicated that the BCAT proteins have additional binding partners governed by their CXXC motif (Coles et al. 2009; Hindy and Conway 2019). These include proteins important in cell signalling and protein folding (e.g. BCAT was identified as a novel chaperone in protein folding through its thiol oxidoreductase activity, El Hindy et al. 2014). Our most recent study offers evidence that these ‘moonlighting’ functions are fundamental in regulating autophagy and play a fundamental role in regulating Aβ (Harris et al. 2020). Here, BCATc-mediated autophagy was shown to be regulated through PKC-mediated phosphorylation, modulated by the redox state of BCATc. Using the SH-SY5Y cell model, we showed that overexpression of BCATc, as observed in AD brain, resulted in Aβ accumulation through an increase in autophagosome synthesis. Importantly, leucine, a key regulator of autophagic flux, significantly reduced BCATc-induced autophagy and consequently Aβ. Our data supports a novel mechanism by which Aβ accumulation may be altered by BCAAs such as leucine, but subject to a more in-depth understanding of nutritional signalling in aging and neurodegenerative conditions such as AD.

## Disorders of BCAA metabolism

### Maple syrup urine disease (MSUD)

Excess or limiting dietary BCAAs cause neurological dysfunction, reducing cognitive function. The autosomal recessive congenital disease, MSUD, is clinically characterised by an accumulation of both BCAAs and their respective α-keto-acid derivatives that are severely toxic to cells (Dancis et al. 1960; Menkes 1959). In untreated cases, infants present with symptoms including neurological dysfunction, seizures and infant death (Chuang et al. 2006). Five classifications of MSUD have been identified (varying from severe classic forms to mild variant types with an additional thiamine responsive form) based on the residual BCKD activity, the age of onset and the concentration of leucine in serum (Chuang and Chuang 2000). Although treatment through restriction of the BCAAs in the diet has most value in milder forms of the condition, patients that are not compliant with their diet or those with more severe forms of the disease are still subject to many side effects.

### Mutations of BCAT leads to mild cognitive impairment and brain lesions

Until recently, mutations of the BCAT gene that manifest clinically were not reported. However, Wang et al. (2015a, b) first reported a case of a 22 year-old male who presented with MCI, lasting longer than 6 years, headaches and a striking blood profile of hypervalinaemia, hyperleucinemia and isoleucinemia, which aligned with a diagnosis of MSUD (Wang et al. 2015a, b). Mutations linked with MSUD were not indicated nor did the patient present with the classical smell of maple syrup from his urine. Although MRI imaging showed associated brain white matter lesions, a feature of MSUD (Jan et al. 2003; Schonberger et al. 2004), brain regions affected in MSUD patients are mainly in the mesencephalon and brain stem as well as thalamus and globus pallidus (Schonberger et al. 2004), whereas in this patient, supratentorial lesions were found without overt abnormalities in these regions. Neuronal damage due to prolonged high brain BCAA concentrations was reported using MRS imaging. Sequencing analysis revealed two heterogeneous mutations including: c.509G> A (p.Arg170Gln) and c.790G>A (p.Glu264Lys). Interestingly, c.509G> A (p.Arg170Gln) was found in his father while c.790G>A (p.Glu264Lys) was found in his mother. Whilst leucine concentrations are usually the highest among the BCAA that are increased in MSUD, valine was shown to be the highest in this case. Moreover, treatment of the patient with vitamin B6 resulted in a sharp decline in valine levels compared with those of leucine and isoleucine. A more recent study identified several additional BCAT2 mutations that ranged between homozygous or compound heterozygous *BCAT2* mutations (Knerr et al. 2019). The authors reported similar biochemical profiles i.e. raised plasma BCAA with wide-ranging cognitive challenges. Other distinguishable features from MSUD such as low‐normal BCKAs with undetectable l‐allo‐isoleucine and absence of acute encephalopathy even with exceptionally high BCAA levels were evident. The mechanisms underlying the brain damage observed in these patients are not well established but will most likely be related to the increased BCAA levels similar to that reported for MSUD (Yudkoff 1997; Barschak et al. 2009; Funchal et al. 2006; Sgaravatti et al. 2003). Decreased transamination would also impact Glu regeneration potentially explaining the MCI presentation.

### Serum BCAA indicators of brain injury

Traumatic brain injury (TBI) is defined as an insult to the brain from an external mechanical force, which can lead to temporary or permanent cognitive impairment dependent on the injury. Following TBI, there is combined metabolic response including Glu toxicity, secondary brain oedema and increased cranial pressure (Vuille-Dit-bille et al. 2012). Interestingly, studies have reported a significant decrease in the plasma BCAAs, whereas the aromatic amino acids (phenylalanine, tryptophan and tyrosine) were increased (Vuille-Dit-bille et al. 2012; Jeter et al. 2013). The BCAAs and aromatic amino acids utilise the same transporters to access the brain. Should the levels of the BCAA decrease then uptake of the aromatic amino acids would exceed that of BCAAs, increasing the synthesis of serotonin and dopamine (Fernstrom and Fernstrom 2007; Scarna et al. 2005). As the BCAA are important nutrient signals in regulating brain Glu, a decrease would signal a reduction in brain repair and dysregulation of glutamate metabolism. In patients with TBI, it was demonstrated that supplementation with BCAA improved cognition and has in some cases improved recovery from a vegetative or minimally conscious state (Aquilani et al. 2008a, 2008). Using an animal model of TBI, Cole et al. (2010) reported improved cognition in rats supplemented with BCAAs following lateral fluid percussion injury. Collectively, these results support a role for BCAA in regulating brain Glu and can impact associated cognitive function. Therefore, additional studies are required to determine the concentration of dietary BCAA that could be beneficial to neurological and neurodegenerative conditions where glutamate toxicity factors.

## Conclusion

Clearly, regulation of brain glutamate is important in AD pathology. Substantial evidence supports a role for ineffective astrocytic clearance of glutamate released during excitation. Moreover, oligomeric Aβ has been shown to interact with glutamate receptors contributing to glutamate excitotoxicity (Hascup and Hascup 2016). Clinically, a role for excitotoxicity in AD pathology is underpinned by the approval of memantine to treat moderate to severe confusion related to AD. Although it does not cure AD it can improve memory, awareness and the ability to carry out day-to-day activities. However, this medication only targets the NMDA receptor, which although reduces excitotoxicity, it does not address other aspects of brain glutamate regulation. As discussed the BCAA and aromatic amino acids are important sources of neurotransmitter synthesis. In disease conditions it is clear that excess (MSUD and BCAT2) or limiting (TBI) amounts of these amino acids result in altered cognitive function. Moreover, mutations in key metabolic enzymes specific to BCAA metabolism, such as BCAT2 and the BCKD complex, result in blood profiles that show BCAAs or BCAA and α-keto acids accumulation, respectively. Treatment through Vitamin B supplementation (BCAT2), BCAA restriction (MSUD), or supplementation (TBI) has improved cognitive outcome. Given that BCAT is increased in key areas of the brain associated with AD pathology and that serum BCAT levels reflect cognitive outcomes it lends weight to further investigate the wider role that BCAT metabolism has on not just glutamate regulation but other pathways associated with AD pathology.

## Abbreviations

KIC: α-Ketoisocaproate
KIV: α-Ketoisovalerate
KMV: α-Keto-β-methylvalerate
AD: Alzheimer’s disease
Aβ: Amyloid β
APP: Amyloid precursor protein
BCAA: Branched chain amino acids
BCAT: Branched chain aminotransferase
BCATc: Cytosolic BCAT
BCATm: Mitochondrial BCAT
BCKA: Branched chain α-keto acids
BCKD: Branched chain α-keto acid dehydrogenase
Cho: Choline
Cr: Creatinine
Glu: Glutamate
Glx: Glu+glutamine
GDH: Glutamate dehydrogenase
MSUD: Maple syrup urine disease
NAA: *N*-acetyl aspartate
NMDAR: *N*-methyl-d-aspartate receptor
NFTs: Neurofibrillary tangles
PLP: Pyridoxal phosphate
PSEN1: Presenilin 1
^1^H MRS: Proton magnetic resonance spectroscopy
mI: Myoinositol
TBI: Traumatic brain injury
